# Older Adolescents Who Did or Did Not Experience COVID-19 Symptoms: Associations with Mental Health, Risk Perception and Social Connection

**DOI:** 10.3390/ijerph18095006

**Published:** 2021-05-09

**Authors:** Jessica Burrai, Benedetta Barchielli, Clarissa Cricenti, Anna Borrelli, Sabina D’Amato, Massimo Santoro, Margherita Vitale, Stefano Ferracuti, Anna Maria Giannini, Alessandro Quaglieri

**Affiliations:** 1 Department of Psychology, Sapienza University of Rome, Via dei Marsi 78, 00185 Rome, Italy; cricenticlarissa@gmail.com (C.C.); annamaria.giannini@uniroma1.it (A.M.G.); alessandro.quaglieri@uniroma1.it (A.Q.); 2 Department of Dynamic and Clinical Psychology, and Health Studies, Sapienza University of Rome, Via degli Apuli 1, 00185 Rome, Italy; benedetta.barchielli@uniroma1.it; 3 Direzione Sanitaria-Servizio di Psicologia Clinica Ospedaliera A. O. U. San Giovanni e Ruggi d’Aragona, Largo Città d’ippocrate, 84131 Salerno, Italy; acquarama@libero.it (A.B.); sabinadamato@yahoo.it (S.D.); dr.massimosantoro@gmail.com (M.S.); margheritavitale79@gmail.com (M.V.); 4 Department of Human Neuroscience, Sapienza University of Rome, Viale dell’Università 30, 00185 Rome, Italy; stefano.ferracuti@uniroma1.it

**Keywords:** self-efficacy, risk-taking, social connectedness, young adult, depression, anxiety, stress

## Abstract

After a decrease in detected cases in the summer, Europe faced the emergence of a second wave of coronavirus disease 19 (COVID-19). Few studies have investigated adolescents, who may constitute a target group with possible lower compliance to public health measures, particularly the social distancing measures. A total sample of 492 participants was included in the study, and the ages of the participants ranged from 18–24 years. According to the hypothesis of our study, the sample was divided into two groups: those who experienced COVID-19 symptoms and those who did not experience COVID-19 symptoms. Demographic characteristics, knowledge, perceptions, and behaviors related to COVID-19 were investigated with ad hoc items; in addition, mood disorders, self-efficacy, and social connectedness were explored. Our results showed significant differences in the variables of risk perception, self-efficacy, and measures of belongingness among older adolescents who did or did not experience COVID-19 symptoms. In this period, adolescents experienced unprecedented disruptions in their daily lives, leading them to isolation and loneliness. Compliance with restrictive measures is considered both a proactive behavior and a social responsibility, especially if supported by prosocial reasons to prevent others from getting sick; therefore, this must be the focus of raising awareness of anti-COVID-19 compliance among adolescents.

## 1. Introduction

At the end of February 2020, Italy faced an outbreak of coronavirus disease 19 (COVID-19), which spread quickly across most of Europe. After a decrease in detected cases in the summer, Europe faced the emergence of a second wave of COVID-19. In this context, the Italian government established a partial lockdown based on a new tiered system, classifying some areas with the highest rates of COVID-19 as high-risk red zones and maintaining preventive measures such as wearing a mask and social distancing. Previous studies on compliance with preventive measures during past epidemics and pandemics (e.g., Ebola virus disease, cholera, avian flu, severe acute respiratory syndrome, equine influenza, and Zika virus) found that factors related to compliance were knowledge of the disease [1,2], perceived effectiveness of the preventive measures [1,3,4], social influence [1], perception of risk [5], concern for self and loved ones, and perceived severity compared to other epidemics [3,6,7]. Different studies have investigated the general population, e.g., [8], psychiatric population, e.g., [9,10], and family patterns, e.g., [11]. However, few studies have investigated adolescents and young adults with ages ranging from a minimum of 15 to a maximum of 22, who may constitute a target group with possible lower compliance to public health measures, particularly with physical distancing measures [12]. However, adolescents had to face new stressors such as the fear of being infected; worrying about their parents’ work (i.e., financial situation); death; restrictions on their privacy; sudden separation from schoolmates, friends, and teachers [13,14,15,16].

These factors were linked to changes in lifestyle habits, increased use of social networks, and changes in eating habits [14,17,18] and experienced emotions, such as loneliness, boredom, and sadness [19,20]; however, adolescents seem to have adapted quickly to the current situation [21].

Furthermore, studies have investigated the impact of COVID-19 on adolescents’ mental health and have found an increase in both pre-existing [22] and non-pre-existing cases of major psychopathological conditions, such as depression, anxiety, and Post-Traumatic Stress Disorder [16,23,24,25,26,27].

On the other hand, studies have investigated the psychosocial environment of adolescents related to pandemic situations. In particular, increased time spent at home with family seems to have influenced conflict with parents, harsh discipline, and parental control [28,29,30]. Regarding relationships with peers and considering the decrease in face-to-face contact, adolescents appear to have increased their communication through social networking, mainly through the use of video calls [21].

During adolescence, the relationship with peers has a relevant influence on adherence to the rules, as well as negative youth-adult relationships [31,32], dysfunctional family functioning (e.g., poor family communication), poor school bonding (poor peer cohesion) [33], low levels of self-control [34], and high sensation-seeking tendencies [35,36]. In contrast, rule compliance appears to be associated with factors, such as the legitimacy of authority, justice [37], and personal factors such as self-efficacy [38].

However, adherence to protective measures during the COVID-19 pandemic was different when considering adults and younger people. Masters and colleagues [39] investigated different generations, from “Generation Z” (18–23 years) to the “Silent Generation” (≥75 years), and showed that the adoption of preventive measures, in particular distancing, increased with age: Generation Z and millennials were the least likely to comply with distancing rules.

One of the reasons that appear to support low adherence to these behaviors is the idea that “there is no alternative,” while the reason that most strongly motivates young people to comply with social distancing is the desire to protect others [40,41]. In addition, young people’s low adherence to COVID-19 rules seems to be associated with low-risk perception, low perception of illness severity, low acceptance of moral rules, low self-control, and peer influence, particularly the relationship with peers who do not comply with the rules [12,41,42,43].

In particular, with the gradual decrease in restrictive measures during the second wave, adolescents considered it no longer necessary to maintain social distancing [43].

Often, young people do not consider COVID-19 a potentially severe disease, and evidence suggests that young people are less vulnerable [44]. Indeed, the period of life between the ages of 10 and 24 years (i.e., adolescence) is often related to increased risk-taking, the need for social connection and peer acceptance, and increased sensitivity to peer influence [42]. However, it seems important to investigate the underlying characteristics of this phenomenon. The likelihood of being infected and experiencing severe COVID-19 symptoms is underestimated by young people, although the spread of COVID-19 might depend on their behaviors. To date, compliance with the rules is still essential to prevent the spread of COVID-19; therefore, the understanding of those factors could mediate the assumption of risky behaviors.

On this basis, we explored the role of social connectedness in maintaining a stable relationship with peers and the dimensions of anxiety, stress, and depression on risk perception and risk behavior in older adolescents (i.e., 18–24 years). Additionally, we explored the difference between the perceived protective behaviors and the actual behaviors to protect oneself against COVID-19 infection. Based on these considerations, the main purpose of our study was to investigate whether the experience of COVID-19 symptoms: (I) influenced risk perception, depression, anxiety and stress; (II) affected self-efficacy in terms of prevention, recognition and home management of COVID-19; and (III) whether the COVID-19 pandemic impacted the sense of belongingness and “being a part of” since the sense of connectedness emerges during adolescence and extends throughout the lifetime.

## 2. Materials and Methods

### 2.1. Participants

A total sample of 492 participants was included in the study. Of the total sample, 27.0% were male, and the ages ranged from 18 to 24 (M = 21.06, SD = 1.82). According to the hypothesis of our study, the sample was divided into two groups: those who experienced COVID-19 symptoms (CE) and those who did not experience COVID-19 symptoms (NCE). The CE sample included 211 participants (18–24 years old; M = 21.07, SD = 1.80), and 21.8% were male. The NCE sample included 281 participants (18–24 years old; M = 21.05, SD = 1.84), and 31.0% were male. With respect to the CE sample, 85.8% of the participants were university students, 3.3% were secondary school students, 7.6% were workers, and 3.3% were unemployed. With regard to the item “Who contracted COVID-19,” 20.9% stated “him/herself,” 14.7% stated “household members,” and 64.5% stated “relatives or close friends.” The full sociodemographic characteristics are described in Table 1.

### 2.2. Procedures

We collected data throughout the Qualtrics Platform online survey. This study was launched on 18 December 2020 and concluded on 5 February 2021. The survey was diffused on social networks and the university’s official website. Participants agreed to participate by signing a digital informed consent form and were informed that the data collection was anonymous and data were not shared outside the current research procedures. After digitally signing the informed consent form, participants were asked to complete the different questionnaires. Demographic characteristics, knowledge, perceptions, and behaviors related to COVID-19 were investigated with ad hoc items; in addition, mood disorders, self-efficacy, and social connectedness were explored. This study was conducted according to the ethical standards of the Helsinki Declaration and was approved by the Institutional Review Board of the Department of Psychology of “Sapienza” University of Rome (protocol number 0002195/18-12-2020).

### 2.3. Materials

The demographic characteristics of sex, age, level of education, and COVID-19 exposure were collected. Subjects were asked to complete the following self-report measures: knowledge related to COVID-19; risk perception of COVID-19; the Depression, Anxiety, and Stress Scale–21 items (DASS-21) [45]; the COVID-19 Prevention, Recognition, and Home-Management Self-Efficacy Scale [46]; the self-reported preventive behavior and motivation to engage in preventive behavior scale [40]; and the Social Connectedness Scale (SCS) [47].

To assess the risk perception of COVID-19, ten items were designed for the purpose of this study, and participants responded to the items using a 5-point Likert scale. In this study, Cronbach’s alpha was 0.66.

For this study, a specific questionnaire with six ad hoc items that evaluated the appropriate COVID-19 coping behaviors was developed. The scale showed minimally acceptable internal consistency with an alpha of 0.68.

Emotional distress was measured by the DASS-21 scale, which contains 21 items measuring three different domains: depression, anxiety, and stress. Depression (e.g., “I felt I was pretty worthless”) includes dysphoria, hopelessness, devaluation of life, self-depression, lack of interest/involvement, anhedonia, and inertia; anxiety (e.g., “I felt I was close to panic”) refers to autonomic nervous system arousal, skeletal musculature effects, situational anxiety, and subjective experience of anxious affects; and stress (e.g., “I felt that I was using a lot of nervous energy”) relates to the presence of nonspecific arousal levels, difficulty relaxing, nervous excitement, irritability, agitation, hyperactivity, and impatience. Participants were asked to respond to questions indicating “how often the situation described has occurred in the last seven days.” All subscales are rated on a 4-point Likert scale ranging from 0 (never) to 3 (almost always). The depression, anxiety, and stress subscales had Cronbach’s alphas of 0.82, 0.74, and 0.85, respectively [48]. In the present study, the Cronbach’s alphas were 0.88 for DASS-Depression, 0.79 for DASS-Anxiety, 0.86 for DASS-Stress, and 0.92 for the total scale.

Two instruments were used to assess self-efficacy. The first was based on previous research on SARS [49], in which a single item was used to investigate “how confident do you feel about avoiding contagion.” This item is based on a 5-point Likert scale ranging from 0 (not confident) to 5 (very confident).

The second instrument was the COVID-19 Prevention, Recognition and Home-Management Self-Efficacy Scale [46]. It contains 19 items “based on the WHO’s recommended behaviors to protect oneself and others from the spread of COVID-19.”

The items are grouped into three categories: (i) Prevention of COVID-19 spread and contagion (e.g., “I do not touch my eyes, nose, or mouth under any circumstances”), (ii) Early recognition of COVID-19 symptoms (e.g., “I identify if I have symptoms of COVID-19 quickly after they appear”), and (iii) Home management of patients with (or suspected) COVID-19 (e.g., “Keep the door to the room of the person with symptoms closed at all times”). Scale response options ranged from 0 (completely sure that I cannot do it) to 100 (completely sure that I can do it). The COVID-19 Prevention, Recognition and Home Management Self-Efficacy Scale had a Cronbach’s alpha of 0.90 and an intraclass correlation coefficient of 0.75. In the present sample, the Cronbach’s alphas were 0.74 for prevention, 0.85 for recognition, 0.88 for home management, and 0.90 for the total scale. The authors reported the internal scoring system as follows: scores below 55 indicated very low self-efficacy, scores ranging from 55 to 68 indicated low self-efficacy, scores ranging from 69 to 82 indicated moderate self-efficacy, scores ranging from 83 to 96 indicated high self-efficacy, and scores above 96 indicated very high self-efficacy.

Self-reported preventive behavior and motivation to engage in preventive behavior were investigated using a single item inspired by the study of [40]. Participants were asked, “In the past 7 days, to what extent did you engage preventive behaviors?”. Six behaviors were presented: washing hands, maintaining social distancing, avoiding crowds, sneezing and coughing safely, wearing a mask, and going out only when allowed. Responses were given on a 5-point scale (1 = not at all to 5 = a great deal).

The SCS [47] measures the level of interpersonal closeness an individual feels in their social world (e.g., friends, peers, and society) and the level of difficulty in maintaining this sense of closeness. It consists of eight items using a 6-point Likert scale (1 = strongly agree to 6 = strongly disagree), and higher scores indicate a greater perceived sense of connectedness (e.g., I feel disconnected from the world around me). The SCS showed a very good internal consistency of 0.91 for social connectedness and an alpha of 0.77 for social assurance. In the present sample, Cronbach’s alphas were 0.92 for social connectedness and 0.79 for social assurance. Cronbach’s alpha of the total scale was 0.82.

## 3. Data Analysis

Statistical analyses were conducted using the Statistical Package for Social Science (SPSS; version 25.0; IBM SPSS, Armonk, NY). First, we tested the internal consistency of the instruments using Cronbach’s alphas, and the results showed internal consistency with an alpha ranging from a minimum of 0.659 to a maximum of 0.924. Descriptive analyses with means and standard deviations were performed. We used independent samples *t*-tests to determine the differences between groups (conducted using SPSS). Pearson correlations were performed to explore the relationships between the main variables. Statistical significance was defined as *p* < 0.05. The distributions of all data were verified for normality. All statistical analyses were performed on de-identified data.

## 4. Results

The analysis of demographic characteristics showed a statistically significant results for sex (*p* < 0.001) and occupation (*p* < 0.001) and no statistically significant result for age (*p* = 0.998). 

With respect to the depression t (490) = −0.271, *p* = 0.787, anxiety t (490) = −1.02, *p* = 0.308 and stress t (490) = −1.554, *p* = 0.121 dimensions of the DASS-21, there were no statistically significant differences between groups; nevertheless, CE groups reported higher overall scores in all three dimensions of the scale compared to the NCE group. With respect to the items on “COVID-19 appropriate behaviors,” there were no statistically significant results between groups.

With respect to the “How confident do you feel about avoiding contagion” item, there was a statistically significant difference between groups (Table 2). The results showed a significantly higher mean in the NCE group (M = 2.95, SD = 0.805) than in the CE group (M = 2.72, SD = 0.738), t (490) = 3.25, *p* < 0.01, d = 0.296.

With regard to the items investigating “Perceived risk of COVID-19”, there was a statistically significant result on “How severe would it be if you contracted COVID-19” (Table 2). The NCE had a higher mean (M = 3.70, SD = 0.762) compared to the CE group (M = 3.50, SD = 0.686), t (490) = 3.04, *p* < 0.01, d = 0.277. The item “How badly do you feel about not being able to meet the people you used to date?” had a statistically significant result (Table 2). The results showed a higher mean in the CE group (M = 4.30, SD = 0.846) than in the NCE group (M = 4.08, SD = 0.997), t (490) = −2.59, *p* < 0.5, d = 0.236. The item “How badly do you feel about having to keep a safe distance from others” yielded a statistically significant result (Table 2). Pairwise comparisons showed a higher mean in the CE group (M = 3.94, SD = 1.08) than in the NCE group (M = 3.62, SD = 1.14), t (490) = −3.17, *p* < 0.01, d = −0.289.

Concerning the COVID-19 Prevention, Recognition and Home-Management Self-efficacy Scale, there was a statistically significant result for recognition of COVID-19 symptoms (Table 2), with a higher mean in the CE group (M = 77.50, SD = 19.71) than in the NCE group (M = 73.22, SD = 22.31), t (477) = −2.21, *p* < 0.05, d = −0.201. Levene’s test indicated unequal variances (F = 3.88, *p* = 0.049); thus, the degrees of freedom were adjusted from 490 to 477. The home management of people with COVID-19 symptoms dimension had a statistically significant result (Table 2) in which the CE group reported a higher mean (M = 80.16, SD = 18.26) than the NCE group (M = 73.61, SD = 20.51), t (490) = −3.67, *p* < 0.001, d = −0.334. The prevention of COVID-19 contagion and spread dimension yielded no statistically significant results between groups, t (490) = −0.179, *p* = 0.858.

The social connectedness scale showed a statistically significant result concerning the “social connectedness” dimension (Table 2). The CE group reported a higher mean (M = 35.29, SD = 8.83) than the NCE group (M = 33.07, SD = 9.88), t (475) = −2.58, *p* < 0.05, d = −0.235. Levene’s test indicated unequal variances (F = 5.69, *p* = 0.017); thus, the degrees of freedom were adjusted from 490 to 475. There was no statistically significant result for the “social assurance” dimension t (486) = 0.753, *p* = 0.452.

### 4.1. Correlations between Variables in the CE Group

Pearson correlations were calculated to explore the relationships between the main variables involved in the study. We found that “How badly do you feel about wearing a mask and not being able to see the full expression on other people’s faces” was significantly and negatively correlated with prevention of COVID-19 contagion, r = −0.174, *p* < 0.05; with “Wash your hands with soap and water or alcohol-based solution,” r = −0.186, *p* < 0.05; and with “Avoid gatherings of large groups of people,” r = −0.166, *p* < 0.05, from the appropriate behavior scale. Additionally, “How badly do you feel about wearing a mask and not being able to see the full expression on other people’s faces” was significantly and positively correlated with the DASS-Depression dimension, r = 0.185, *p* < 0.01.

We found that DASS-Depression scores were significantly and negatively correlated with the “social connectedness” dimension, r = −0.564, *p* < 0.01; and the “social assurance” dimension, r = −0.208, *p* < 0.01. DASS-Anxiety dimension scores were significantly and negatively correlated with the “social connectedness,” r = −0.318, *p* < 0.01; and with the “How confident do you feel about avoiding contagion,” r = −0.202, *p* < 0.01. DASS-Stress scores were significantly and negatively correlated with “How confident do you feel about avoiding contagion”, r = −0.164, *p* < 0.05; “social connectedness,” r = −0.325, *p* < 0.01; and “social assurance,” r = −0.165, *p* < 0.05.

### 4.2. Correlations between Variables in the NCE Group

We found that “How badly do you feel about wearing a mask and not being able to see the full expression on other people’s faces” was significantly and negatively correlated with the “social assurance” dimension, r = −0.229, *p* < 0.01; and significantly and positively correlated with the DASS-Anxiety, r = 0.171, *p* < 0.01, and DASS-Stress dimensions, r = 0.211, *p* < 0.01. We found that DASS-Depression was significantly and negatively correlated with the “social connectedness” dimension, r = −0.515, *p* < 0.01; the “social assurance” dimension, r = −0.307, *p* < 0.01; the “Recognition of COVID-19 symptoms,” r = −0.205, *p* < 0.01; and with the “Home management of people with COVID-19 symptoms,” r = −0.253, *p* < 0.01. The DASS-Anxiety dimension was significantly and negatively correlated with the “social connectedness” dimension, r = −0.346, *p* < 0.01; and with the “social assurance” dimension r = −0.245, *p* < 0.01. DASS-Stress dimension was significantly and negatively correlated with the “Recognition of COVID-19 symptoms,” r = −0.132, *p* < 0.01; “Home management of people with COVID-19 symptoms,” r = −0.184, *p* < 0.01; the “social connectedness” dimension, r = −0.387, *p* < 0.01; and the “social assurance” dimension, r = −0.216, *p* < 0.01.

## 5. Discussion

The main goal of this study was to better understand the condition of older adolescents during the long-term COVID-19 pandemic, considering that the extraordinary measures adopted by national governments to face the pandemic had unprecedented effects on adolescents’ daily lives. In this regard, social distancing and isolation strategies have been the worldwide primary measures to prevent the risk of infection [50]. Although these measures benefit the entire community, they lead to stress, anxiety, and a sense of helplessness in everyone; for this reason, their psychological effects cannot be overlooked [51].

Our results showed significant differences in the variables of risk perception, self-efficacy, and measures of belongingness among older adolescents who experienced (CE) or did not experience (NCE) COVID-19.

With respect to the DASS-21 questionnaire, the CE and NCE groups showed no statistically significant differences. However, measures of distress along the three axes revealed “moderate” depression and “mild” anxiety for both the CE and NCE groups, “moderate” stress for the CE group and “mild” stress for the NCE group. The lack of a significant difference reported in this study could be explained by the fact that COVID-19 has long-term effects (e.g., the future perspective becomes confusing, fearful, uncertain, and distressing). In this regard, the risk and fear of contagion have changed social and interpersonal relationships, socialization opportunities, education and training systems, and physical activities, especially for younger people; furthermore, home confinement leads to uncertainty and anxiety in both children and adolescents [52]. This is true regardless of whether adolescents had direct COVID-19 experience.

Furthermore, the CE group showed a higher mean difference than the NCE group on social connectedness (one of two measures of belongingness, based on H. Kohut’s [53] self-psychology theory), which focuses on the emotional distance or connection between the self and other people. Social connectedness concerns those aspects of belonging that Kohut [53] described as an “intense and pervasive sense of security” and a sense of being “human among humans.” We can hypothesize that having experienced COVID-19 may increase the proximity (interest in health status) of friends and relatives; for this reason, this may have an impact on “feeling part of something.”

Regarding the second measure of belongingness (i.e., social assurance), no differences between groups were found. With respect to the correlation analysis conducted, the DASS-21 depression dimension showed a negative correlation with both measures of belongingness (social connectedness and social assurance) for both the CE and NCE groups. Likewise, the anxiety and stress dimensions of the DASS-21 showed a negative correlation with social connectedness and social assurance for both the CE and NCE groups. Therefore, we can hypothesize that as anxiety, stress and depression increase, older adolescents feel more emotionally distant from friends, relatives, and society and feel less a “part of something.” These data seem to be explained by prolonged isolation due to restricted home confinement, forced removal from school friends and relatives, confusing or contradictory communication on the pandemic, and the uncertainties of personal and family futures that may have led to increased anxious, stressed, and depressed responses.

Moreover, older NCE adolescents had a higher mean on the dimension of avoiding COVID-19 infection (i.e., “How confident do you feel about avoiding contagion”) and scored higher than CE adolescents on the severity of being infected with COVID-19 (i.e., “How severe would it be if you contracted COVID-19”). Indeed, higher perceived self-efficacy to take preventive measures is associated with greater perceived severity of the COVID-19 disease. These data agree with the literature as risk perception is significantly related to COVID-19 severity and coronavirus self-efficacy [54]; furthermore, this result seems to agree with evidence suggesting young people are less vulnerable to the effects of COVID-19 [44]. These differences could be explained by previous knowledge of COVID-19’s consequences in the CE group. The NCE group did not have direct experience with COVID-19 and therefore did not know what to expect. The NCE group perceives itself to be more effective at avoiding COVID infection as it has not yet had any experience and feels “protected” from the risk of infection. The results just discussed are also in line with further scientific literature. Indeed, people who believe that they are more vulnerable perceive a higher risk of infection and fear the virus and are also more likely to adopt preventive behaviors. This suggests that developing people’s ability to cope with the impact of COVID-19 may increase the adoption of preventive behaviors. These findings suggest that risk perception, along with other factors, may influence the levels of individuals’ preventive behaviors [1]. 

In addition, the CE group showed higher levels of bad moodiness than the NCE group and, in particular, with some measures of prevention of COVID-19 infection (e.g., “How badly do you feel about not being able to meet the people you used to date” and “How badly do you feel about having to keep a safe distance from others”). Therefore, we assumed that the CE group could consider COVID-19 infection as less serious and be more bothered by mandatory avoidance behaviors.

This is confirmed by a strong positive correlation found in the CE group between the item “How badly do you feel about wearing a mask and not being able to see the full expression on other people’s faces” and the depression dimension of the DASS-21 and strong negative correlations with the COVID-19 contagion prevention item and appropriate COVID-19 coping behaviors (i.e., “Wash your hands with soap and water or alcohol-based solution” and “Avoid gatherings of large groups of people”). Thus, we can hypothesize that since CE individuals consider COVID-19 to be less severe, they may experience more discomfort in strictly adhering to protective measures and that having direct experience with the disease may have generated a false perception of invulnerability.

With respect to the COVID-19 Prevention, Recognition and Home-Management Self-efficacy Scale, the older adolescent CE group had higher means for symptom recognition and home management of COVID-19. These data are quite clear; indeed, those who experienced COVID-19 felt more capable of recognizing the symptoms of infection (e.g., fever or chills, cough, shortness of breath or difficulty breathing, fatigue, muscle or body aches, headache, loss of taste or smell, etc.) and in the home management of those infected with COVID-19 including the following: avoiding all contact with other family members, sleeping alone and staying in a dedicated room, limiting movement in house’s spaces in order to avoid meeting other people, periodically measuring oxygen saturation, taking medication to control symptoms, etc. Such differences could be explained by the information that is available to an individual on COVID-19. The NCE group has less information (only the information generally acquired from media), so they may feel less effective at handling the consequences of COVID-19. Although the Protection Motivation Theory (PMT) states that self-efficacy is considered a robust predictor of various health-related behaviors [55,56], no significant differences were found between the CE and NCE groups in appropriate COVID-19 behaviors as measured by different survey items (e.g., “Always keep a distance of at least three feet between myself and others”). This can be explained by the coping-appraisal process (i.e., one of the three processes of PMT), which suggests coping as the sum of evaluations of the effectiveness and self-efficacy of responses minus any physical or psychological “costs” of adopting the recommended preventive response. Thus, our results, having been collected during the second wave, may reflect the habit of implementing certain types of protective behaviors regardless of having had experience with COVID-19.

The results showed strong correlations between “Recognition of COVID-19 symptoms” and “Home management of people with COVID-19 symptoms” and the stress and depression dimensions of the DASS in the NCE group. Self-efficacy seems to influence people’s perception of themselves and may often be inconsistent with reality, resulting in a self-image that is too positive or too negative.

Stress is related to low self-efficacy, that is, beliefs about the inability to master new or challenging tasks, perform a particular behavior, or exercise control over events [57,58]. In the present study, people’s self-efficacy in the prevention, detection of symptoms, and home management of COVID-19, when they had not experienced COVID-19, may have triggered a pattern in which perceived inefficacy is closely related to stress and depression. In fact, self-efficacy is an important dispositional resource that mitigates threat appraisals, state anxiety, and cortisol secretion [59].

These results offer an opportunity to reflect on the population examined here since young people tend to frequent crowded places; if they do not comply with the health measures recommended by the government, they risk spreading COVID-19. These results could highlight the importance of prosocial aspects in the management of preventive measures in adolescents for social policies. The importance of active involvement in understanding compliance with anti-COVID rules, rather than the imposition of enforcement, emerges.

However, the study has some limitations. First, it is limited by the use of online surveys and the use of self-reported questionnaires, which may have influenced the findings through well-known biases, including method biases and social desirability biases. Additionally, the study was limited by the relatively small sample size and was conducted using the online convenience sampling strategy without random sample selection. In addition, the study could not investigate comparisons between CE and NCE (i.e., regarding sex and education level) due to numerically significant differences in the sample, and using an observational design limits the generalizability of the results.

## 6. Conclusions

To the best of our knowledge, this study is the first to provide data on a population of older adolescents who have or have not experienced COVID-19. To prevent the spread of COVID-19, adolescents experienced unprecedented disruptions in their daily lives, leading them to isolation and loneliness. Adolescence is a stage of life in which excitement and risk-taking are experienced; thus, some adolescents may feel invulnerable and not follow guidelines regarding distancing and personal hygiene. These factors mean that adhering to social distancing rules can be especially difficult for youth and must be assertively addressed with adolescents.

Valuing adolescents’ peer support system is essential. For them, it can be very important and helpful to talk to their peers about their feelings and common problems they face. It has also been found that among adolescents, compliance with restrictive measures is considered both a proactive behavior and a social responsibility, especially if supported by prosocial reasons to prevent others from getting sick; this must be the focus of raising awareness of anti-COVID-19 compliance among adolescents.

## Figures and Tables

**Table 1:** Demographic characteristics of the studied population.

Descriptive Statistics			
		CE	NCE
Sex	Male	46	87
	Female	165	194
	Total	211	281
Age	Mean	21.07	21.05
	Std. Deviation	1.80	1.84
	Range	18–24	18–24
Occupation	Unemployed	7	4
	Employed	16	8
	Second-undergraduate degree	7	16
	University degree	181	253
	Total	211	281
Who have contracted the COVID-19	Him/her-self	44	-
	Household members	31	-
	Relatives or close friends	136	-
	Total	211	-
Sport Activities	Yes	21	33
	No	190	248
	Total	211	281
Friends Meeting	Yes	80	82
	No	131	199
	Total	211	281

CE = individuals with COVID-19 symptoms experience; NCE = individuals without COVID-19 experiences.

**Table 2:** Comparison between groups (ANOVAs).

ANOVA							
Item	t	df	Sig.	d	Multiple Comparisons	M	SE
How confident do you feel about avoiding contagion?	3.25	490	0.01 **	0.296	NCE vs. CE	0.230	0.071
Recognition of COVID-19 symptoms.	−2.215	477	0.05 *	−0.201	CE vs. NCE	−4.28	1.93
Home management of people with COVID-19.	−3.671	490	0.001 ***	−0.334	CE vs. NCE	−6.54	1.78
Social connectedness.	−2.582	475	0.05 *	−0.235	CE vs. NCE	−2.22	0.861
How severe would it be if you contracted COVID-19?	3.041	490	0.01 **	0.277	NCE vs. CE	0.202	0.067
How badly do you feel about not being able to meet the people you used to date?	−2.586	490	0.05 *	−0.235	CE vs. NCE	−0.220	0.085
How badly do you feel about having to keep a safe distance from others?	−3.172	490	0.01 **	−0.289	CE vs. NCE	−0.324	0.102

* *p* < 0.05; ** *p* < 0.01; *** *p* < 0.001; CE = individuals with COVID-19 symptoms experience; NCE = individuals without COVID-19 experience. M = Mean; SE = Standard error.

## Data Availability

The data presented in this study are available on request from the corresponding author. The data are not publicly available due to privacy considerations.
